# Directed Synthesis of {Mn18Cu6} Heterometallic Complexes**

**DOI:** 10.1002/anie.201208781

**Published:** 2013-01-10

**Authors:** Victoria A Milway, Floriana Tuna, Andrew R Farrell, Laura E Sharp, Simon Parsons, Mark Murrie

**Affiliations:** WestCHEM, School of Chemistry, University of GlasgowGlasgow G12 8QQ (UK); EPSRC National UK EPR Facility, School of Chemistry and Photon Science Institute, The University of ManchesterManchester M13 9PL (UK); EaStCHEM School of Chemistry, The University of EdinburghEdinburgh, EH9 3JJ (UK)

**Keywords:** copper, heterometallic complexes, magnetic properties, manganese, polynuclear clusters

The development of new synthetic strategies to assemble high-nuclearity transition metal complexes is a key target in modern coordination chemistry.^1^ One of the driving forces for this is their fascinating magnetic properties for example, single-molecule magnets^2^ or magnetic refrigerants^3^ and molecules with large spin ground states^4^ or large anisotropy barriers.^5^ The use of two, or more, different metal ions to assemble these clusters is an attractive synthetic target and controlling the bottom-up assembly of large heterometallic molecules is a considerable challenge.^6^, ^7^ However, the potential rewards are significant, as there is a real possibility of control/design over the individual magnetic parameters that contribute to the overall molecular properties.^8^ Furthermore, new functionality can be added, such as the combination of magnetic and optical properties,^9^ or the production of catalysts or catalyst precursors with high activity and/or selectivity.^10^

Previously, polydentate ligands with specific binding sites/donor atoms,^11^ linear linkers such as cyanide^12^ or rigid structure-directing ligands^13^ have been used to prepare heterometallic complexes. Herein, we describe a new step-by-step approach to synthesize large 3d–3d′ heterometallic oxo-bridged clusters. Firstly, we use a preformed Cu^II^ complex, which contains multiple, latent hydroxy binding sites, to target the trapping and encapsulation of an inner metal-oxo core. Secondly, the choice of Cu^II^ as the central ion increases the flexibility further, due to its range of typical coordination environments from [4] to [4+2]. We report two compounds that contain a striking “core-shell” {Mn_18_Cu_6_} complex as either a hexa- or dication, where the Cu^II^ precursors encapsulate a hexacapped cuboctahedral manganese oxide {Mn^III^_12_Mn^II^_6_O_14_} nanocluster.

The Cu^II^ center is enclosed using the bis-tris propane ligand {2,2′-(propane-1,3-diyldiimino)bis[2-(hydroxymethyl)propane-1,3-diol] (H_6_L, Scheme 1) forming the precursor complex [Cu(H_6_L)Cl]Cl⋅1.25 H_2_O (**1**⋅1.25 H_2_O) (see Supporting Information, Figure S1) in almost quantitative yield (see Experimental). This is then redissolved and utilized in a second reaction to generate the heterometallic complexes: addition of base to a solution of **1**, followed by addition of MnCl_2_⋅4 H_2_O leads to the formation of [Mn_18_Cu_6_O_14_(H_2_L)_6_Cl_2_(H_2_O)_6_]Cl_6_⋅H_2_O (**2**⋅H_2_O) using NMe_4_OH/EtOH or [Mn_18_Cu_6_O_14_(H_2_L)_6_Cl_6_]Cl_2_⋅10 H_2_O⋅6 CH_3_OH (**3**⋅10 H_2_O⋅ 6 CH_3_OH) using NEt_3_/MeOH. Both compounds can be prepared reproducibly, albeit in low yields, which is not uncommon in the area of high-nuclearity complexes.^14^ We have been unable to obtain these complexes using a range of one-pot reactions and preformation of the Cu^II^ complex appears to be essential.

**Scheme 1:**
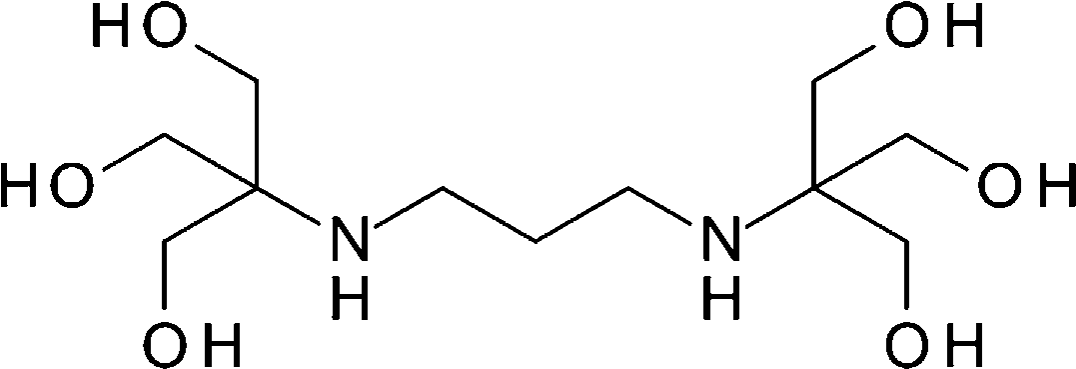
Bis-tris propane (H_6_L).

The structure of the cationic cluster in **2** is based upon a {Mn^III^_12_Mn^II^_6_O_14_}^20+^ core, encapsulated by six {Cu(H_2_L)}^2−^ groups. Oxidation states have been confirmed by bond-valence sum (BVS) calculations and by consideration of charge balance/coordination environments. The twelve Mn^III^ and fourteen O^2−^ anions, form a hollow cube (ca. 3.8 Å O–O edge) (Figure 1 a). The Mn^III^ cations describe a cuboctahedron, capped on each square face by a Mn^II^, forming a giant octahedron (Figure 1 b). Six faces of this giant octahedron are capped by a Cu^II^ center, which resides off-center, above one of the smaller constituent {Mn^II^Mn^III^_2_} triangular faces (Figure 1 c). The Cu^II^ ions describe a further octahedron, twisted with respect to the {Mn^II^_6_} octahedron, giving a remarkable level of self-assembly: polyhedral shells of expanding size describing archimedian {Mn^III^_12_} < platonic {Mn^II^_6_} < platonic {Cu^II^_6_} solids (Figure 2).

**Figure 1:**
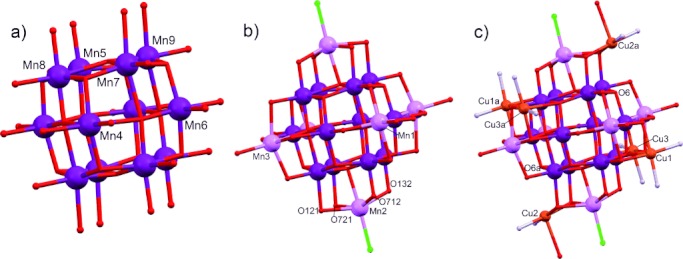
POV-Ray depictions of a) the {Mn^III^_12_O_14_} core of **2**, b) expansion to include the Mn^II^ octahedron encapsulating the core, c) the overall heterometallic core of **2**. Mn^III^, purple; Mn^II^, pink; Cu^II^, bronze; Cl, green; O, red (oxide=capped stick, alkoxide=ball and stick); N, blue (C and H atoms omitted for clarity).

**Figure 2:**
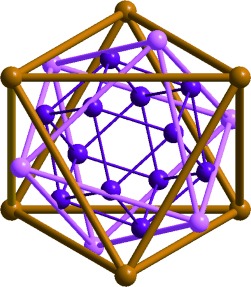
Expanding polyhedral shells {Mn^III^_12_} < {Mn^II^_6_} < {Cu^II^_6_} in **2** (colors as previously described).

The outer (final) coordination site of each Mn^II^ center is occupied by either a terminal water {Mn(1), Mn(3) and symmetry equivalent (s.e.)} or chloride ligand {Mn(2) and s.e.} (Figure 1 b). Each H_2_L^4−^ ligand displays the same bonding mode η^3^:η^3^:η^2^:η^2^:η^1^:η^1^:μ_6_ (Figure 3) and each Cu^II^ center is bridged to a Mn^II^ and two Mn^III^ centers via two μ_3_ ligand alkoxide arms (Figures 1 c and 3). The Cu^II^ centers are best described as distorted [4+1] coordinate, with the apical bond (ca. 2.6 Å) to either a core oxide anion {for Cu(1), Cu(3)} or an (outer) water ligand {for Cu(2)}. Hence, four {CuN_2(eq)_O_2(eq)_O_(ax)_} pyramids point towards the core and two point away (Figure 4) and the coordinative flexibility of the Cu^II^ center (i.e. the direction of the Cu^II^ axial bonds) modulates the shape of the {Mn_18_Cu_6_} complex. For Cu(1) and Cu(3) there is an additional (outer) weak interaction with a lattice chloride anion {2.9808(1), 2.959(3) Å} and for Cu(2) an additional (inner) weak interaction with a cube corner oxide anion {O(6), 2.986(3) Å} (Figure 1 c).

**Figure 3:**
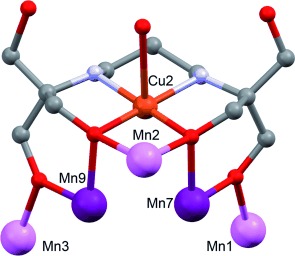
H_2_L^4−^ ligand binding mode in **2**; the apical bond of Cu2 is pointing outwards from the core, to a H_2_O ligand. Colors are as previously described, plus C, gray (H atoms not shown).

**Figure 4:**
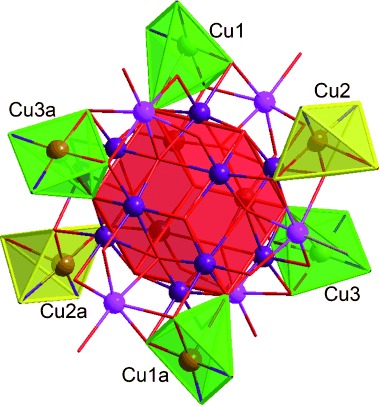
Differing coordination environments of the Cu^II^ cations; yellow pyramids indicate Cu^II^ centers where the apical bond points away from the core, green pyramids have the apical Cu^II^ bond pointing to the core. Red cube represents the core Mn^III^-oxide cube. Cu1a=Cu1{1.5−*x*, 0.5−*y*, 1−*z*}.

Compound **3** contains a similar {Mn_18_Cu_6_} complex, as a dication, where the core structure is largely the same as for compound **2** (Figure S2 and Tables S5 and S6). However, in this case, each Mn^II^ center is equivalent and has a terminal chloride ligand (cf. either Cl^−^ or H_2_O in **2**) (Figure 5). If we describe each Cu^II^ center as [4+1] as in **2**, then each apical ligand (ca. 2.6 Å) bonds to a core oxide anion (cf. two of these were to an {outer} water ligand in **2**, that is, pointing away from the core). These structural changes result in a more compact core with higher symmetry (*S*_6_). If we describe the Cu^II^ centers as distorted [4+2] instead, the second axial position is occupied by the oxygen atom of a CH_2_OH ligand arm on an adjacent molecule (Cu1–O73′ ca. 2.75 Å) (Figure S3) and each {Mn_18_Cu_6_} cluster is connected to six nearest neighbor clusters via double {Cu-OCCN-Cu′} bridges (Cu⋅⋅⋅Cu′ ca. 6.2 Å), creating a 3D network (Figure 6, S4). Hence, the subtle change to the reaction conditions also induces a new level of self-assembly of the {Mn_18_Cu_6_} complexes in **3**; this is not possible in **2**, due to the presence of the two centers (Cu2 and s.e.) with H_2_O ligands.

**Figure 5:**
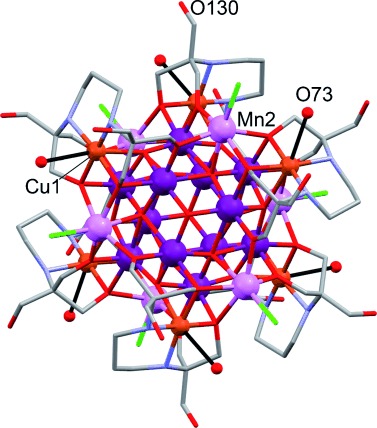
**3**, viewed along the three-fold axis. Bonds to oxygen atoms of neighboring clusters, utilized in forming a 3D net are shown as solid black lines.

**Figure 6:**
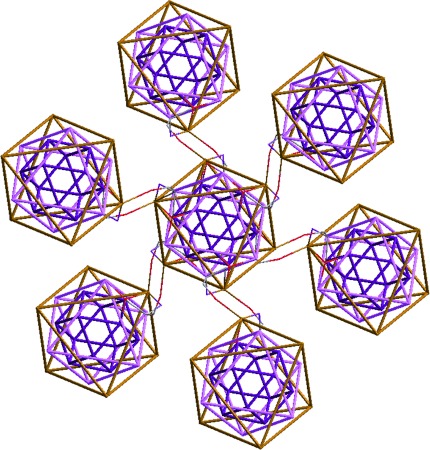
Pseudo-octahedral arrangement of {Mn_18_Cu_6_} clusters in the extended structure, linked by Cu-OCCN-Cu bridges.

The largest heterometallic 3d-based cluster is a mixed-valent {Cu^I^_4_Cu^II^_13_Mn^II^_4_Mn^III^_12_Mn^IV^_12_} cluster, prepared from Cu powder, Mn(OAc)_2_ and triethanolamine in DMF.^15^ Further high-nuclearity Mn/Cu complexes include: {Mn^II^Cu^II^_8_} and {Mn^II^_5_Cu^II^_4_}^11^ or {Mn^III^_6_Cu^II^_10_} and {Mn^III^_8_Mn^IV^_4_Cu^II^_8_}.^16^ However, none of these one-pot reactions result in either similar metal ion topologies or oxidation levels to those found in **2** and **3**. Interestingly, the {Mn^III^_12_} core structure of the {Mn_18_Cu_6_} complexes is related to the smaller Mn-oxo clusters, [Mn^IV^Mn^III^_6_Mn^II^_6_O_8_(OEt)_6_(O_2_CPh)_12_]^17^ and [Mn^II^Mn^III^_12_(μ_4_-O)_8_(μ_4_-Cl)_6_(*t*Bu-PO_3_)_8_]^18^ where the central position is occupied by a Mn^IV^ or a Mn^II^ cation, respectively (cf. empty in **2** and **3**). Hence, our approach may provide a more general route to trap and build upon stable metal-oxo core architectures: here trapping a {Mn^III^_12_} core and adding {Mn^II^_6_} and {Cu^II^_6_} shells.

Structurally closest to **2** and **3** is perhaps the polyoxometalate anion [Ti_12_Nb_6_O_44_]^10−^, which also has an empty central cavity, in which Ti^IV^ and Nb^V^ take the place of Mn^III^ and Mn^II^, respectively.^19^ Comparisons can also be drawn with Pd^0^ clusters: [Pd_23_(CO)_20_(PEt)_10_] consists of a centered cuboctahedral {Pd_13_} core, with square faces capped by Pd atoms in the sites occupied by the six Mn^II^ in {Mn_18_Cu_6_}.^20^ The resulting {Pd_19_} giant octahedron is capped on four of its eight faces by additional Pd atoms, in positions close to those occupied by Cu^II^ in **2** and **3**.

The core of **2** is observed by ESI-MS (Figure S5, Table S7). All labile aquo ligands are lost and ion-pairs are observed for {[Cu^II^_6_Mn^II^_6_Mn^III^_12_(H_2_L)_6_O_14_Cl_2_]Cl_3_]}^3+^ (*m*/*z* 1146.7) and {[Cu^II^_6_Mn^II^_6_Mn^III^_12_(H_2_L)_6_O_14_Cl_2_]Cl_4_]}^2+^ (*m*/*z* 1737.5) and some fragmentation of the parent ion is observed, [Cu(H_5_L)]^+^ (*m*/*z* 344.1). The solution stability provides further potential for using these reaction systems to probe heterometallic cluster assembly.

The bridging in **2** is complex and each metal cation is bridged to between three and eight others, via single oxygen bridges. Bridging angles range from 88.28–107.83°. The overall picture is similar for **3**, except that the higher symmetry of the molecule results in a minimum of four bridging connections to neighboring metal ions. The majority of the bridging angles are large, and as a result, we would expect antiferromagnetic coupling to dominate; which proves to be the case (Figure S6). Magnetization vs. field data (Figure S7) suggests a large number of excited states with similar energies, and a poorly defined ground state. AC measurements do not show any evidence of frequency dependence; this is unsurprising as the Jahn–Teller axes of the Mn^III^ centers are nearly perpendicular, leaving little net magnetic anisotropy.

Using our step-by-step approach, starting with a preformed Cu^II^ complex, we can trap and encapsulate manganese oxide nanoclusters. Reactions changing the anion (Cl^−^), precursor (Cu^II^) and core metal ion (Mn) are all underway, in order to assemble new heterometallic clusters and to explore the self-assembly of high-nuclearity complexes.

## Experimental Section

All reagents and solvents were obtained from commercial suppliers and used without further purification.

Synthesis of **1**: H_6_L (5.70 g, 20.2 mmol) and CuCl_2_⋅2 H_2_O (5.28 g, 31.0 mmol) were combined in ethanol (120 mL) and heated to 60 °C. A dark green solution formed, followed by precipitation of a pale blue solid (15 mins). The mixture was heated for 5 h. After cooling, the blue precipitate [Cu(H_6_L)Cl]Cl⋅1.25 H_2_O (**1**⋅1.25 H_2_O) was collected by filtration and washed with ethanol. Yield 8.63 g, 96 %. IR: 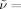
3140, 3018, 2943, 1470, 1428, 1307, 1263, 1114, 1077, 1064, 1011, 761 cm^−1^. Elemental analysis (C_11_H_26_N_2_O_6_CuCl_2_⋅1.25 H_2_O) [%], found: C 30.01, H 6.50, N 6.38; calcd: C 30.07, H 6.54, N 6.38. Single crystals suitable for X-ray study were obtained by recrystallization from ethanol yielding **1**⋅0.5 EtOH (see Supporting Information).

Synthesis of **2**: **1**⋅1.25 H_2_O (109 mg, 0.263 mmol) was dissolved in hot ethanol (60 mL at 60 °C). NMe_4_OH⋅5 H_2_O (166 mg, 0.842 mmol) was added, and immediately dissolved, resulting in a clear royal blue solution, which was heated for 30 min. MnCl_2_⋅4 H_2_O (166 mg, 0.837 mmol) was added, resulting in an immediate color change to dark gray, followed by formation of a precipitate. The mixture was heated for 4 h, and the precipitate (108 mg) was removed by filtration. Black crystals of [Mn_18_Cu_6_O_14_(H_2_L)_6_Cl_2_(H_2_O)_6_]Cl_6_⋅H_2_O formed in the filtrate over 1 month (ca. 6 mg, 4 %) (see Supporting Information). IR: 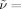
3351, 3262, 3212, 2921, 2864, 1634, 1455, 1426, 1393, 1260, 1154, 1100, 1079, 1027, 925, 791, 761 cm^−1^. Elemental analysis (C_66_H_144_N_12_O_56_Cu_6_Mn_18_Cl_8_, **2**) [%], found: C 21.81, H 4.08, N 4.60; calcd: C 21.72, H 3.97, N 4.61. MS (ESI^+^, *m*/*z*): 344.1, 1146.7, 1737.5 (see Table S7).

Synthesis of **3**: **1**⋅1.25 H_2_O (108 mg, 0.259 mmol) was dissolved in methanol (30 mL). NEt_3_ (0.07 mL, 0.502 mmol) was added, and immediately dissolved, resulting in a clear royal blue solution which was stirred at ambient temperature for 30 min. MnCl_2_⋅4 H_2_O (110 mg, 0.557 mmol) was added, resulting in an immediate color change to blue-black. The solution was heated for 3 h, and filtered. Black crystals of [Mn_18_Cu_6_O_14_(H_2_L)_6_Cl_6_]Cl_2_⋅10 H_2_O⋅6 CH_3_OH formed in the filtrate over 3 weeks (ca. 4 mg, 3 %) (see Supporting Information). IR: 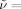
3361, 3234, 3215, 2947, 2868, 1622, 1458, 1429, 1390, 1262, 1156, 1101, 1084, 1061, 1026, 940, 932, 793, 720 cm^−1^. Elemental analysis (C_66_H_144_N_12_O_50_Cu_6_Mn_18_Cl_8_⋅4.5H_2_O⋅4CH_3_OH, **3**⋅4.5 H_2_O⋅ 4 CH_3_OH) [%], found: C 22.39, H 4.59, N 4.62; calcd: C 22.38, H 4.21, N 4.47. A further 17 mg microcrystalline black solid was collected from the solution after removal of the single crystals. IR, as above. Elemental analysis (**3**⋅4.5 H_2_O⋅4 CH_3_OH) [%], found: C 22.31, H 4.59, N 4.60. Total yield; 21 mg, 18 %.

CCDC 907989 http://www.ccdc.cam.ac.uk/cgi-bin/catreq.cgi(**1**), 907990 http://www.ccdc.cam.ac.uk/cgi-bin/catreq.cgi(**2**), and 907991 http://www.ccdc.cam.ac.uk/cgi-bin/catreq.cgi(**3**) contain the supplementary crystallographic data for this paper. These data can be obtained free of charge from The Cambridge Crystallographic Data Centre via http://www.ccdc.cam.ac.uk/data_request/cif.

## Supplementary material

As a service to our authors and readers, this journal provides supporting information supplied by the authors. Such materials are peer reviewed and may be re-organized for online delivery, but are not copy-edited or typeset. Technical support issues arising from supporting information (other than missing files) should be addressed to the authors.
