# Supplementary material for: Directed Synthesis of {Mn18Cu6} Heterometallic Complexes
Source: Angew Chem Int Ed Engl. 2013 Jan 10;52(7):1949–52. doi: 10.1002/anie.201208781 (PMC3601430; doi:10.1002/anie.201208781)
Supplement: Supplementary file 1 [file anie0052-1949-SD1.pdf]

Angewandte Chemie
Eine Zeitschrift der Gesellschaft Deutscher Chemiker

Supporting Information

© Wiley-VCH 2013

69451 Weinheim, Germany

Directed Synthesis of {$Mn_{18}Cu_6$} Heterometallic Complexes**

*Victoria A. Milway, Floriana Tuna, Andrew R. Farrell, Laura E. Sharp, Simon Parsons, and Mark Murrie**

anie_201208781_sm_miscellaneous_information.pdf

Supporting Information

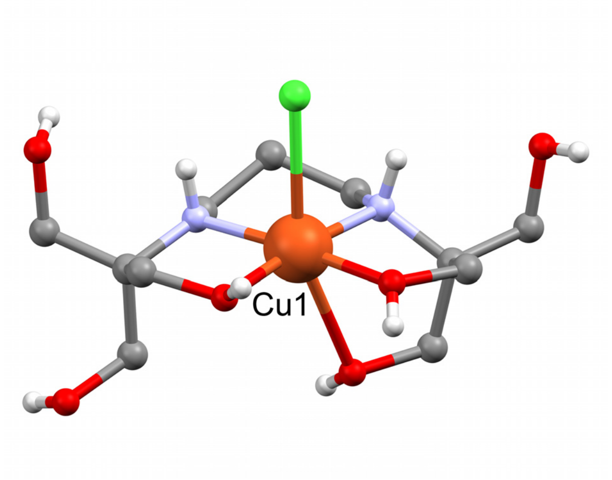

Figure S1: POV-Ray image of $[Cu(H_6L)Cl]^+$. CH protons removed for clarity. Cu, bronze; C, grey; N, blue; O, red; Cl, green. Cu-N, 1.998(3)-2.002(3); Cu-O_{eq}, 1.958(2)-1.976(3); Cu-O_{ax}, 2.585(3); Cu-Cl, 2.734(1) Å.

Table S1: Bond distances (Å) in (**2**):

Cu1—Cl1	2.981 (3)	Mn4—O1	2.305 (3)
Cu1—O5	2.637 (3)	Mn4—O3	1.925 (3)
Cu1—O111	1.941 (4)	Mn4—O4	1.938 (3)
Cu1—N21	1.979 (5)	Mn4—O7	1.930 (3)
Cu1—N61	1.961 (5)	Mn4—O731	2.178 (3)
Cu1—O711	1.949 (4)	Mn4—O732	1.920 (3)
Cu2—O9	2.571 (6)	Mn5—O1	1.927 (3)
Cu2—N22	2.000 (5)	Mn5—O4	1.945 (3)
Cu2—N62	2.009 (5)	Mn5—O5	1.937 (3)
Cu2—O121	1.956 (4)	Mn5—O711	2.176 (4)
Cu2—O721	1.943 (4)	Mn5—O712	1.914 (3)
Cu3—C13	2.854 (5)	Mn5—O2	2.340 (8)
Cu3—O7	2.593 (3)	Mn6—O3	2.279 (3)
Cu3—N23	1.976 (4)	Mn6—O1	1.926 (3)
Cu3—N63	1.970 (5)	Mn6—O5	1.923 (3)
Cu3—O131	1.944 (3)	Mn6—O6	1.937 (3)
Cu3—O731	1.943 (3)	Mn6—O111	2.177 (4)
Mn1—O8	2.209 (4)	Mn6—O112	1.936 (3)
Mn1—O111	2.241 (4)	Mn7—O1	2.334 (3)
Mn1—O711	2.230 (4)	Mn7—O2	1.920 (3)
Mn1—O722	2.129 (4)	Mn7—O6	1.923 (3)
Mn1—O732	2.104 (4)	Mn7—O7	1.945 (3)
Mn2—O712	2.162 (3)	Mn7—O721	2.200 (3)
Mn2—Cl2	2.3971 (17)	Mn7—O722	1.940 (4)
Mn2—O2	2.273 (3)	Mn8—O5	1.951 (3)
Mn2—O121	2.236 (4)	Mn8—O2	2.318 (3)
Mn2—O721	2.296 (4)	Mn8—O3	1.935 (3)
Mn2—O132	2.128 (4)	Mn8—O7	1.918 (3)
Mn2—O712	2.162(4)	Mn8—O131	2.148 (3)
Mn3—O122	2.161 (3)	Mn8—O132	1.920 (3)
Mn3—O112	2.093 (4)	Mn9—O3	2.360 (3)
Mn3—O3	2.264 (3)	Mn9—O4	1.941 (3)
Mn3—O10	2.225 (4)	Mn9—O2	1.921 (3)
Mn3—O131	2.294 (3)	Mn9—O6	1.922 (3)
Mn3—O731	2.144 (4)	Mn9—O121	2.186 (3)
		Mn9—O122	1.935 (3)

Table S2: Bond angles (°) in (**2**):

O5-Cu1-N21	103.40(18)	O2-Mn2-O721	77.49(12)	O3-Mn6-O112	83.11(13)
O5-Cu1-O711	78.12(13)	O2-Mn2-O121	77.84(12)	O1-Mn6-O5	83.64(13)
O5-Cu1-O111	82.11(12)	O2-Mn2-O132	78.62(12)	O1-Mn6-O111	87.17(14)
Cl1-Cu1-N61	84.52(16)	O712-Mn2-O2	79.66(12)	O3-Mn6-O6	88.08(13)
Cl1-Cu1-N21	85.31(17)	O721-Mn2-O132	90.99(14)	O1-Mn6-O6	88.99(13)
N21-Cu1-O111	86.3(2)	O712-Mn2-O132	91.42(14)	O111-Mn6-O112	89.37(14)
N61-Cu1-O711	87.2(2)	O712-Mn2-O121	96.30(13)	O5-Mn6-O112	92.89(14)
O111-Cu1-O711	88.81(15)	Cl2-Mn2-O121	98.23(11)	O6-Mn6-O112	95.28(14)
Cl1-Cu1-O711	92.76(12)	O712-Mn2-Cl2	98.78(10)	O5-Mn6-O111	96.04(14)
Cl1-Cu1-O111	93.61(12)	O10-Mn3-O131	100.05(14)	O6-Mn6-O111	97.16(14)
N21-Cu1-N61	97.7(2)	O122-Mn3-O10	100.82(14)	O1-Mn7-O2	100.04(12)
O5-Cu1-N61	99.07(17)	O112-Mn3-O10	104.76(14)	O7-Mn7-O721	103.02(13)
O9-Cu2-O121	100.30(19)	O131-Mn3-O731	75.34(12)	O1-Mn7-O6	78.33(12)
O9-Cu2-N62	82.1(2)	O3-Mn3-O131	76.37(11)	O2-Mn7-O6	83.65(14)
O121-Cu2-O721	86.26(15)	O3-Mn3-O731	77.74(12)	O1-Mn7-O722	84.61(13)
N62-Cu2-O721	87.28(17)	O112-Mn3-O3	80.13(12)	O1-Mn7-O7	86.52(12)
O9-Cu2-N22	87.3(2)	O122-Mn3-O3	82.01(12)	O721-Mn7-O722	87.27(14)
N22-Cu2-O121	87.67(18)	O112-Mn3-O131	91.69(13)	O2-Mn7-O721	87.64(13)
O9-Cu2-O721	95.63(18)	O122-Mn3-O112	92.29(13)	O2-Mn7-O7	89.73(14)
N22-Cu2-N62	98.7(2)	O122-Mn3-O731	92.58(13)	O6-Mn7-O721	93.35(13)
O7-Cu3-N63	102.01(15)	O10-Mn3-O731	96.94(14)	O7-Mn7-O722	93.61(14)
O7-Cu3-O131	79.44(12)	O1-Mn4-O3	101.96(13)	O6-Mn7-O722	94.40(15)
O7-Cu3-O731	81.89(12)	O1-Mn4-O4	79.46(12)	O5-Mn8-O131	101.93(13)
Cl3-Cu3-N23	83.86(14)	O1-Mn4-O732	82.72(13)	O2-Mn8-O7	79.53(13)
Cl3-Cu3-N63	85.42(14)	O3-Mn4-O7	83.00(13)	O2-Mn8-O132	81.77(13)
N63-Cu3-O731	85.69(17)	O3-Mn4-O731	84.58(13)	O3-Mn8-O7	83.03(14)
N23-Cu3-O131	86.38(17)	O1-Mn4-O7	87.72(12)	O3-Mn8-O131	87.10(13)
O131-Cu3-O731	88.59(14)	O3-Mn4-O4	90.01(13)	O5-Mn8-O2	87.37(13)
Cl3-Cu3-O131	92.70(12)	O731-Mn4-O732	90.86(14)	O5-Mn8-O3	88.47(14)
Cl3-Cu3-O731	94.22(11)	O7-Mn4-O732	92.28(15)	O131-Mn8-O132	90.59(14)
N23-Cu3-N63	99.2(2)	O7-Mn4-O731	94.16(13)	O7-Mn8-O131	92.50(13)
O7-Cu3-N23	99.29(15)	O4-Mn4-O732	95.80(15)	O7-Mn8-O132	93.36(15)
O8-Mn1-O732	103.89(15)	O4-Mn4-O731	99.69(13)	O5-Mn8-O132	95.65(15)
O8-Mn1-O722	104.55(14)	O2-Mn5-O1	101.22(12)	O2-Mn8-O3	99.95(12)
O111-Mn1-O711	75.00(13)	O4-Mn5-O711	105.26(14)	O3-Mn9-O2	101.41(12)
O1-Mn1-O711	76.77(12)	O2-Mn5-O4	77.87(13)	O4-Mn9-O121	105.12(14)
O1-Mn1-O111	77.70(12)	O2-Mn5-O712	83.15(13)	O3-Mn9-O4	78.20(12)
O1-Mn1-O732	79.55(12)	O1-Mn5-O5	83.25(13)	O2-Mn9-O6	83.65(14)
O1-Mn1-O722	81.94(12)	O1-Mn5-O711	85.79(14)	O3-Mn9-O122	84.48(13)
O722-Mn1-O732	89.83(14)	O2-Mn5-O5	87.06(13)	O3-Mn9-O6	86.12(13)
O111-Mn1-O722	91.49(14)	O1-Mn5-O4	89.52(13)	O2-Mn9-O121	86.96(13)
O711-Mn1-O732	95.37(14)	O711-Mn5-O712	89.75(14)	O121-Mn9-O122	87.06(14)
O8-Mn1-O711	96.19(14)	O5-Mn5-O711	90.99(14)	O4-Mn9-O2	89.15(14)
O8-Mn1-O111	98.20(15)	O4-Mn5-O712	93.46(14)	O6-Mn9-O121	91.95(14)
Cl2-Mn2-O721	103.47(10)	O5-Mn5-O712	94.98(14)	O4-Mn9-O122	94.17(14)
Cl2-Mn2-O132	105.68(11)	O3-Mn6-O1	100.02(13)	O6-Mn9-O122	94.73(14)
O121-Mn2-O721	72.02(13)	O3-Mn6-O5	79.89(13)	O3-Mn6-O112	83.11(13)
O5-Cu1-N21	103.40(18)	O2-Mn2-O721	77.49(12)		

Table S3: Metal-Metal distances in (**2**):

Cu1—Mn1	3.1331 (10)
Cu1—Mn5	3.2232 (10)
Cu1—Mn6	3.0979 (10)
Cu2—Mn2	3.1600 (10)
Cu2—Mn9	3.4358 (9)
Cu3—Mn3	3.1191 (9)
Cu3—Mn4	3.1094 (9)
Cu3—Mn8	3.1533 (9)
Mn1—Mn4	3.2407 (10)
Mn1—Mn5	3.2338 (10)
Mn1—Mn7	3.2191 (10)
Mn2—Mn5$^{ii, a}$	3.2677 (10)
Mn2—Mn7	3.2394 (10)
Mn2—Mn9	3.2048 (10)
Mn3—Mn6^{ii}	3.2128 (10)
Mn3—Mn9^{ii}	3.2349 (10)
Mn3—Mn4	3.2139 (10)
Mn3—Mn8	3.2434 (10)
Mn4—Mn9^{ii}	3.0260 (10)
Mn4—Mn5	2.9990 (10)
Mn4—Mn7	3.0462 (10)
Mn4—Mn8	2.8851 (10)
Mn5—Mn9^{ii}	3.0347 (10)
Mn5—Mn8^{ii}	3.0551 (10)
Mn5—Mn6	2.8765 (10)
Mn6—Mn8^{ii}	2.9970 (10)
Mn6—Mn7	3.0195 (10)
Mn6—Mn9	3.0394 (10)
Mn7—Mn8	2.9908 (10)
Mn7—Mn9	2.8609 (10)

a) e.g. Mn5^{ii} = Mn5{1.5-x, 0.5-y, 1-z}

Table S4: Metal-metal bridging angles(°) in (**2**):

Cu1-O5-Mn5	88.24(12)	Mn4-O731-Mn3	96.07(13)
Cu1-O5-Mn6	84.05(12)	Mn5-O1-Mn6	96.58(14)
Cu1-O5-Mn8	166.23(16)	Mn5-O2-Mn7	166.05(17)
Cu3-O7-Mn4	85.53(11)	Mn5-O2-Mn9	90.25(13)
Cu3-O7-Mn7	166.24(16)	Mn5-O4-Mn4	101.12(14)
Cu3-O7-Mn8	87.34(12)	Mn5-O4-Mn9	102.68(15)
Mn1-O1-Mn4	90.02(12)	Mn5-O5-Mn6	96.37(14)
Mn1-O1-Mn5	100.21(14)	Mn5-O711-Cu1	102.63(16)
Mn1-O1-Mn6	100.06(14)	Mn6-O111-Cu1	97.42(15)
Mn1-O1-Mn7	88.53(11)	Mn6-O3-Mn4	166.56(17)
Mn1-O111-Cu1	96.78(15)	Mn6-O3-Mn8	90.28(13)
Mn1-O111-Mn6	93.95(13)	Mn6-O3-Mn9	81.84(10)
Mn1-O711-Cu1	96.92(15)	Mn6-O6-Mn7	102.94(15)
Mn1-O711-Mn5	94.42(14)	Mn6-O6-Mn9	103.96(15)
Mn1-O722-Mn7	104.47(15)	Mn7-O1-Mn5	168.11(17)
Mn1-O732-Mn4	107.20(16)	Mn7-O1-Mn6	89.74(12)
Mn2-O121-Cu2	97.61(15)	Mn7-O6-Mn9	96.16(15)
Mn2-O132-Mn8	107.72(16)	Mn7-O7-Mn4	103.64(15)
Mn2-O2-Mn5	90.18(12)	Mn7-O7-Mn8	101.43(15)
Mn2-O2-Mn7	100.85(13)	Mn7-O721-Cu2	110.20(16)
Mn2-O2-Mn8	90.91(11)	Mn7-O721-Mn2	92.16(13)
Mn2-O2-Mn9	99.31(13)	Mn8-O131-Cu3	100.69(15)
Mn2-O712-Mn5	106.42(15)	Mn8-O131-Mn3	93.73(12)
Mn2-O721-Cu2	96.03(15)	Mn8-O2-Mn5	81.97(11)
Mn3-O112-Mn6	105.71(15)	Mn8-O2-Mn7	89.28(13)
Mn3-O122-Mn9	104.18(15)	Mn8-O2-Mn9	167.19(17)
Mn3-O131-Cu3	94.43(13)	Mn8-O3-Mn4	96.71(14)
Mn3-O3-Mn4	99.88(14)	Mn8-O5-Mn5	103.57(15)
Mn3-O3-Mn6	90.01(12)	Mn8-O5-Mn6	101.36(15)
Mn3-O3-Mn8	100.84(14)	Mn9-O121-Cu2	111.97(17)
Mn3-O3-Mn9	88.76(11)	Mn9-O121-Mn2	92.87(13)
Mn3-O731-Cu3	99.37(14)	Mn9-O2-Mn7	96.29(15)
Mn4-O1-Mn5	89.80(12)	Mn9-O3-Mn4	89.24(12)
Mn4-O1-Mn6	166.88(18)	Mn9-O3-Mn8	167.61(17)
Mn4-O1-Mn7	82.09(10)	Mn9-O4-Mn4	102.53(15)
Mn4-O7-Mn8	97.13(14)	Cu1-Cl1-Cu1	162.98(10)
Mn4-O731-Cu3	97.78(14)		

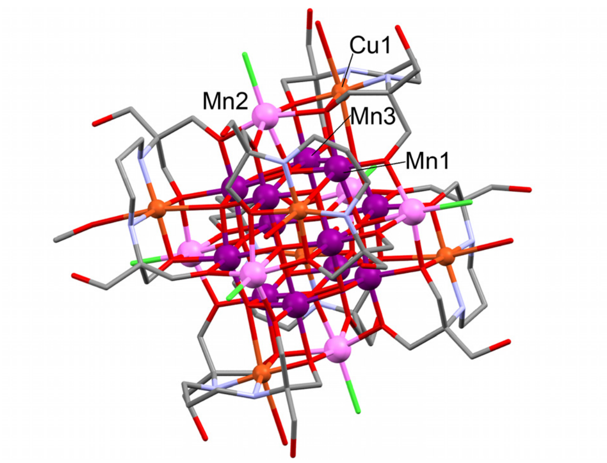

Figure S2: **3**; H atoms, lattice solvent, and anions removed for clarity. C = grey; O = red; N = blue; Cl = green; Mn^{III} = purple; Mn^{II} = pink; Cu = orange. All Cu^{II} are bound to an O^{2-} of the core.

Table S5: Bond distances (Å) in (**3**):

Cu1-O1	2.566(4)	Mn2-Cl1	2.423(2)
Cu1-O11	1.964(3)	Mn2-O2	2.307(3)
Cu1-N2	1.961(6)	Mn2-O11	2.287(4)
Cu1-N6	1.979(3)	Mn2-O71	2.185(3)
Cu1-O71	1.941(4)	Mn2-O72	2.110(5)
Cu1-O73	2.766(4)	Mn2-O120	2.133(3)
Mn1-O1	1.921(3)	Mn3-O1	1.945(3)
Mn1-O2	1.913(4)	Mn3-O2	1.935(3)
Mn1-O11	2.190(4)	Mn3-O3	1.919(2)
Mn1-O120	1.929(4)	Mn3-O71	2.188(3)
Mn1-O1a	1.946(3)	Mn3-O72	1.920(3)
Mn1-O2a	2.309(3)	Mn3-O2a	2.322(5)

Table S6: Bond angles (°) in **3**:

O1-Cu1-O11	80.1(1)	Cl1-Mn2-O2	173.57(9)
O1-Cu1-N2	98.8(2)	Cl1-Mn2-O11	99.6(1)
O1-Cu1-N6	105.5(1)	Cl1-Mn2-O71	98.8(1)
O1-Cu1-O71	84.9(1)	Cl1-Mn2-O72	104.5(1)
O1-Cu1-O73	168.4(1)	Cl1-Mn2-O120	105.1(1)
O11-Cu1-N2	88.2(2)	O2-Mn2-O11	75.7(1)
O11-Cu1-N6	171.1(2)	O2-Mn2-O71	75.9(1)
O11-Cu1-O71	88.0(1)	O2-Mn2-O72	79.3(1)
O11-Cu1-O73	88.6(1)	O2-Mn2-O120	79.8(1)
N2-Cu1-N6	97.7(2)	O11-Mn2-O71	74.6(1)
N2-Cu1-O71	174.1(2)	O11-Mn2-O72	153.3(1)
N2-Cu1-O73	83.5(2)	O11-Mn2-O120	93.5(1)
N6-Cu1-O71	85.7(1)	O71-Mn2-O72	90.3(1)
N6-Cu1-O73	85.4(1)	O71-Mn2-O120	154.9(1)
O71-Cu1-O73	91.9(1)	O72-Mn2-O120	91.3(2)
O1-Mn1-O2	83.9(1)	O1-Mn3-O2	82.7(1)
O1-Mn1-O11	91.5(1)	O1-Mn3-O3	163.3(2)
O1-Mn1-O120	92.6(1)	O1-Mn3-O71	96.1(1)
O1-Mn1-O1	162.9(2)	O1-Mn3-O72	91.2(1)
O1-Mn1-O2	78.9(1)	O1-Mn3-O2	86.9(1)
O2-Mn1-O11	86.5(1)	O2-Mn3-O3	90.5(2)
O2-Mn1-O120	173.6(1)	O2-Mn3-O71	83.9(1)
O2-Mn1-O1	89.0(1)	O2-Mn3-O72	171.5(1)
O2-Mn1-O2	100.5(1)	O2-Mn3-O2	102.7(1)
O11-Mn1-O120	88.2(1)	O3-Mn3-O71	98.3(2)
O11-Mn1-O1	103.5(1)	O3-Mn3-O72	96.9(2)
O11-Mn1-O2	167.4(1)	O3-Mn3-O2	79.8(2)
O120-Mn1-O1	95.8(2)	O71-Mn3-O72	90.8(1)
O120-Mn1-O2	84.0(1)	O71-Mn3-O2	173.1(1)
O1-Mn1-O2	87.2(1)	O72-Mn3-O2	82.9(1)

Table S6: Metal-metal distances (Å) and Metal-metal bridging angles (°) for **3**:

Cu1-Mn2	3.162(2)	Cu1-O1-Mn3	83.3(1)
Cu1-Mn3	3.035(1)	Cu1-O1-Mn1	166.4(2)
Cu1-Mn1	3.174(1)	Mn2-O11-Mn1	93.7(2)
Cu1-Mn1	4.4817(8)	Mn2-O71-Mn3	97.6(2)
Mn2-Mn1	3.267(1)	Mn2-O120-Mn1	105.8(2)
Mn2-Mn3	3.291(1)	Mn2-O72-Mn3	107.9(2)
Mn2-Mn3	3.261(1)	Mn2-O2-Mn1	101.1(2)
Mn2-Mn1	3.242(1)	Mn2-O2-Mn1	89.2(1)
Mn1-Mn3	4.208(1)	Mn2-O2-Mn3	89.6(1)
Mn1-Mn1	3.003(2)	Mn2-O2-Mn3	101.4(2)
Mn1-Cu1	4.482(1)	Mn3-O1-Mn1	103.4(2)
Mn1-Mn1	3.003(1)	Mn3-O1-Mn1	96.3(2)
Mn1-Mn3	4.2116(9)	Mn3-O3-Mn3	101.6(2)
Mn1-Mn3	3.054(1)	Mn3-O2-Mn3	88.1(1)
Mn1-Mn3	2.8801(9)	Mn3-O2-Mn1	167.0(2)
Mn3-Mn3	2.973(2)	Mn3-O2-Mn1	82.5(1)
Mn3-Mn3	2.973(1)	Mn3-O2-Mn1	96.9(2)
Mn3-Mn1	4.212(1)	Mn3-O2-Mn1	165.8(2)
Cu1-O71-Mn2	99.9(2)	Mn1-O2-Mn1	90.2(1)
Cu1-O11-Mn2	95.8(2)	Mn1-O1-Mn1	101.9(2)
Cu1-O1-Mn1	88.8(1)		

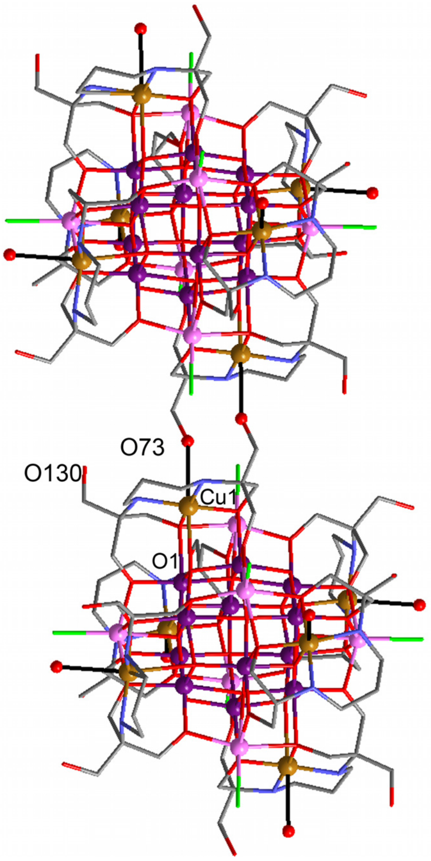

Figure S3: Intermolecular Cu-OCCN-Cu bridge between clusters in **3.**

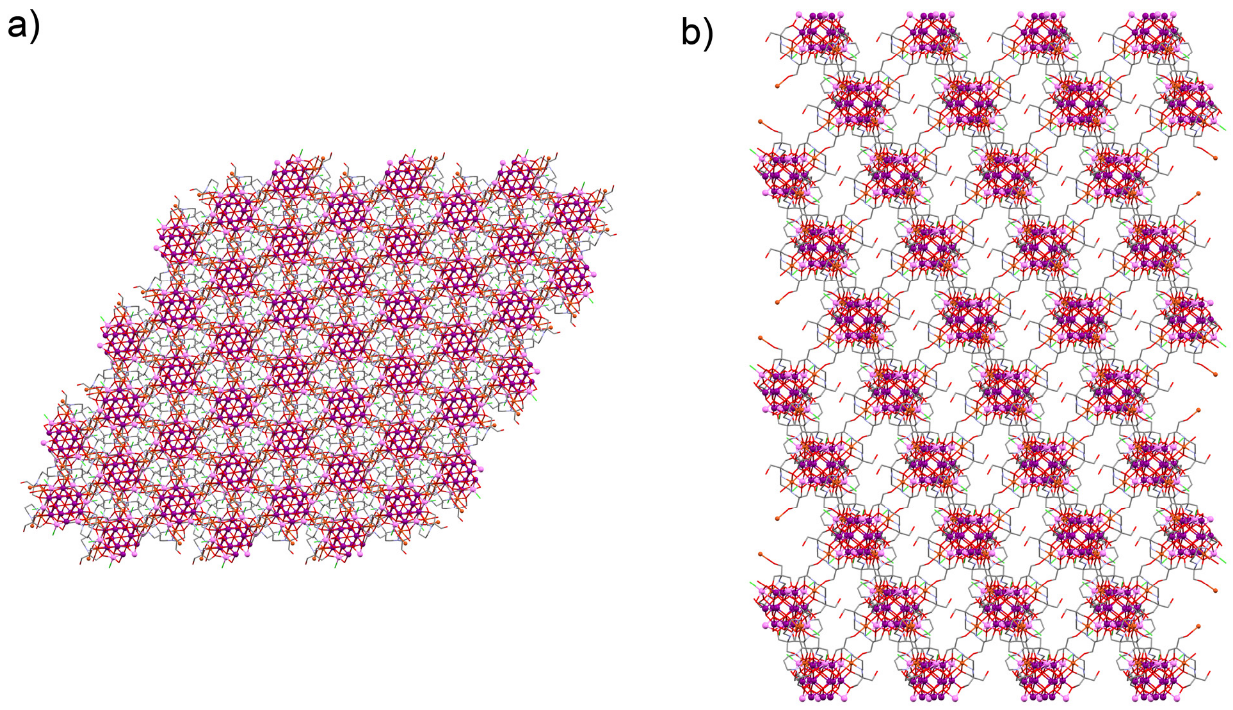

Figure S4: Extended structure of **3** viewed along a) the *c* axis and b) the *a* axis.

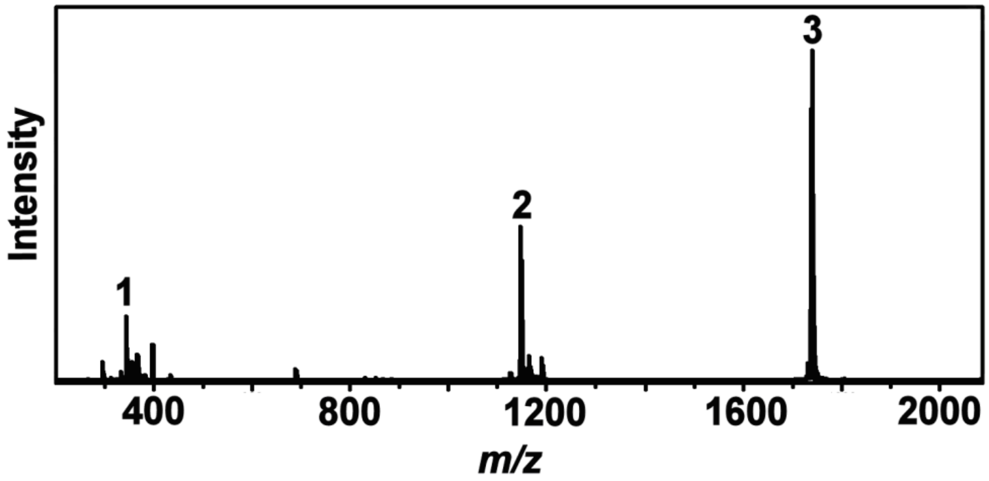

Figure S5: ESI^+ spectrum for **2.**

Table S7: Peak assignment for **2**

Assignment	*m/z*	formula
**1**	344.1	$[Cu^{II}(C_{11}H_{25}N_2O_6)]^+$
**2**	1146.7	$\{[Cu^{II}_6Mn^{II}_6Mn^{III}_{12}(C_{11}H_{22}N_2O_6)_6O_{14}Cl_2]Cl_3]\}^{3+}$
**3**	1737.5	$\{[Cu^{II}_6Mn^{II}_6Mn^{III}_{12}(C_{11}H_{22}N_2O_6)_6O_{14}\ Cl_2]Cl_4]\}^{2+}$

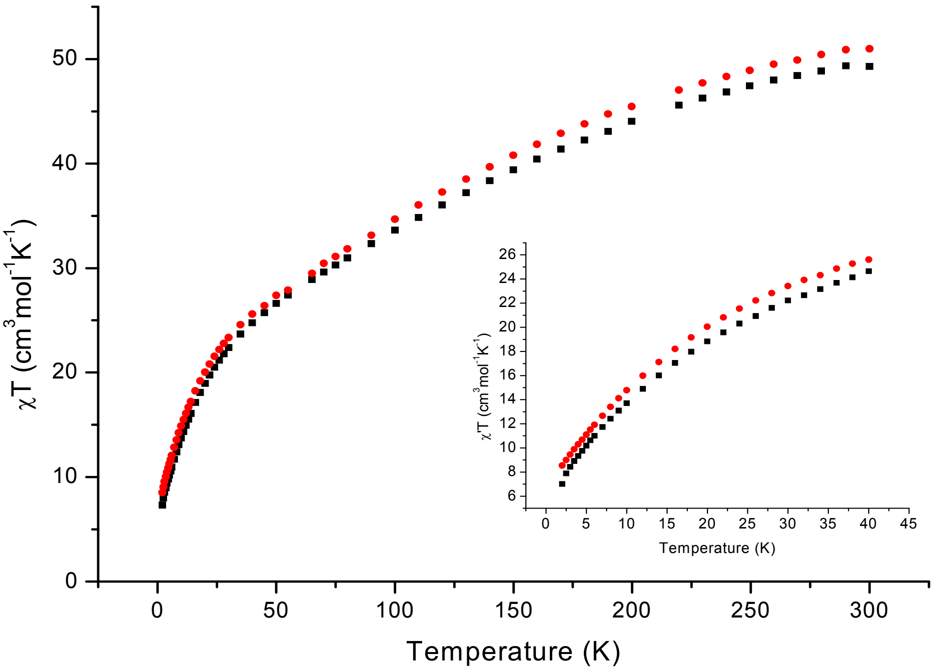

Figure S6: Temperature dependence of χT for **2** (black) and **3** (red) from 300 − 2 K measured in a field of 1 kOe. Inset shows temperature dependence of χ'T from 40-2 K, at a frequency of 10 Hz, and static field of 0 G.

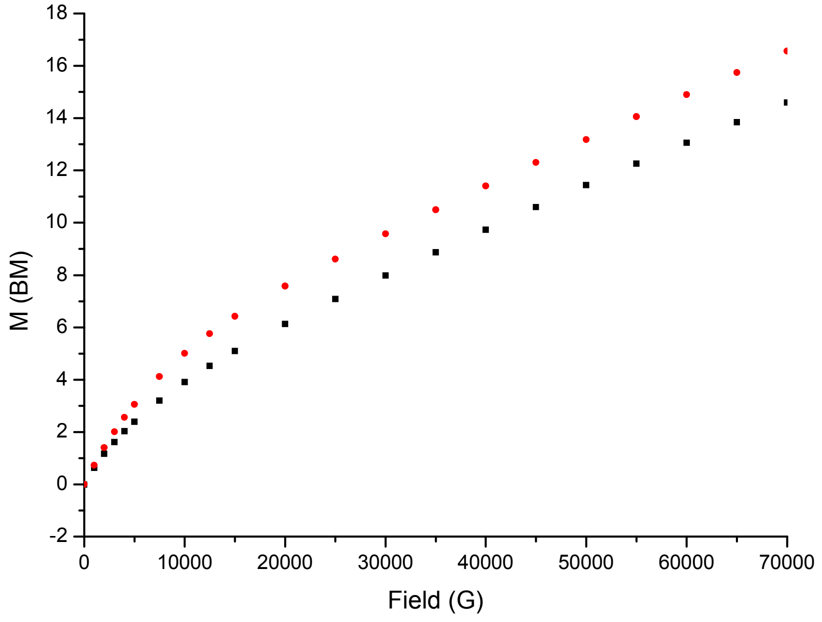

Figure S7: Magnetization data for **2** (black) and **3** (red) at 2 K measured from 0-7 T.

Experimental:

Crystallography:

Summary of crystallographic data for **1**: monoclinic, *C*2/*c*, $C_{11}H_{26}N_2OCuCl_2 \cdot 0.5(CH_3CH_2OH)$, *a* = 29.224 (4), *b* =10.5279 (12), *c* = 12.8908 (17) Å, β = 115.720 (8)°, V = 3573.1 (8), Z= 8, T = 100 K, M = 439.82 g/mol, ρ = 1.637 g/cm^3, MoKα radiation (λ=0.71073 Å), R_1 = 0.032 for 217 parameters and 1882 unique reflections with $I > 2.0\sigma$, and wR2 = 0.073 for all 39220 reflections.

Summary of crystallographic data for **2**: monoclinic, *C*2/*c*, $C_{66}H_{144}N_{12}O_{56}Cu_6Mn_{18}Cl_8 \cdot H_2O$ *a* = 24.1549 (6) Å, *b* = 22.4730 (6), *c* = 24.0784 (6), β = 100.312 (1), V = 12859.4 (6), Z= 4, T = 100 K, M = 3653.33 g/mol, ρ = 1.887 g/cm^3, MoKα radiation (λ=0.71073 Å), Refinement on F; R_1 = 0.052 for 757 parameters and 9100 reflections with $I > 2.0\sigma$.

Summary of crystallographic data for **3**: trigonal, *R*-3, $C_{66}H_{144}N_{12}O_{50}Cu_6Mn_{18}Cl_8 \cdot 10H_2O \cdot 6CH_3OH$, a = 19.7840 (8) Å, *c* = 33.4938 (14), γ = 120°, V = 11353.3 (8), Z= 3, T = 100 K, M = 3920.00 g/mol, ρ = 1.557 g/cm^3, MoKα radiation (λ=0.71073 Å), R_1 = 0.049 for 237 parameters and 3 restraints and 3383 unique reflections with $I > 2.0\sigma$, and wR2 = 0.162 for all 4318 reflections.

All crystallographic intensity data were collected using a Bruker APEX2CCD diffractometer equipped with graphite-monochromated Mo-Kα radiation (λ = 0.71073 Å) and an Oxford Cryosystems low-temperature device. The structure of **1** was solved by direct methods using SIR92[1] and refined using full-matrix least-squares refinement on F^2 using CRYSTALS.[2] The structure of **2** was solved using SUPERFLIP[3,4] and refined using full-matrix least-squares refinement on F using CRYSTALS. The structure of **3** was solved using SIR92 and refined using full-matrix least-squares refinement on F^2 using SHELXL.[5]

For **1**, all non-H atoms were refined anisotropically. H atoms attached to C atoms were added geometrically. H atoms bonded to N and O were found in a difference map. H atoms were refined as riding on their parent atoms, apart from those on the disordered ethanol of crystallisation which were not refined.

For **2**, as many atoms as possible in the primary structure were refined anisotropically, as was one of the lattice chloride anions. Several atoms in the complex exhibited large adps and it was decided that due to disorder inherent to the structure it was more sensible to model these with isotropic displacement parameters. The other lattice chloride anions and the water of crystallisation are also refined isotropically. H atoms attached to C atoms were added geometrically, H atoms on N and O atoms were found in a difference map. They were refined as riding on their parent atoms. Hydroxyl H atoms on some of the non-bridging CH_2OH arms were added in calculated positions.

For **3**, during initial refinement it was noted that R1 was unreasonably large, and so the ROTAX algorithm was used to search for possible merohedral twins. A twinned domain, related to the first domain by [1 0 0 -1 -1 0 0 0 -1], was found. The two domains exist in approx. 68.5 %/ 31.5% ratio. Later on during the refinement, evidence was found for disordered solvent of crystallisation between clusters. It proved difficult to model this with a conventional molecular model and so it was decided to apply the SQUEEZE routine implemented within PLATON. This cannot be performed on twinned data, and so the data were de-twinned using an undocumented LIST 6 instruction. This also corrects for anomalous dispersion and so the scattering factors in the SHELXL instruction file were modified to account for this. SQUEEZE has accounted for 621 electrons per cell, or 207 per formula unit, since Z=3. We tentatively assign this as 6 MeOH and 10 H_2O molecules of crystallisation, since this is consistent with evidence from elemental analysis. Most non-H atoms in the main cluster are refined with adps, with the exception C1, C120/C121, C130/C131, O120/O121and O130/O131 which are refined isotropically. Lattice Cl2 is also refined anisotropically. Similarity restraints were applied to the disordered ligands. The atom O120/O121 could have been modelled on a single site, however for ease of application of similarity restraints we split it into the two half-occupied atoms described, and used EADP/EXYZ restraints to ensure that the two components have identical thermal and positional parameters. H atoms were placed in calculated positions using appropriate HFIX instructions and refined using AFIX instructions. The occupancy of Cl2 was refined freely to approximately 1/3 then fixed at 1/3. Considering site multiplicity this gives a sum total of 2 Cl per cluster.

Magnetic studies:

Magnetic measurements in the temperature range 1.8 - 300 K were performed on polycrystalline samples constrained in eicosane, using a Quantum Design SQUID magnetometer equipped with a 7 T magnet. Data were corrected for the diamagnetism of the compounds by using Pascal's constants and for the diamagnetic contributions of the sample holder and eicosane by measurements. AC susceptibility data were collected on cooling with an ac drive field of 1.55 G oscillating at frequencies between 1 and 1200 Hz.

Mass Spectral data:

Mass spectrometry data were obtained using a Bruker MicrOTOF-Q in ESI positive ion mode. The following parameters were used for the MS scans; The calibration solution used was Agilent ES tuning mix solution, Recorder No. G2421A, enabling calibration between approximately 100 m/z and 3000 m/z. This solution was diluted 60:1 with MeOH. The sample was dissolved in MeOH (10-5 M) and introduced into the MS via direct injection at 180 μL/h, with the dry gas temperature set to 180 °C. The ion polarity for all MS scans recorded was positive, with the voltage of the capillary tip set at 4500 V, end plate offset at -500 V, funnel 1 RF at 300 Vpp and funnel 2 RF at 400 Vpp, hexapole RF at 400 Vpp, ion energy 5.0 eV, collision energy at 10 eV, collision cell RF at 500 Vpp, transfer time at 100.0 μs and the pre-pulse storage at 10.0 μs. The spectrum was collected for 2 mins.

References:

[1] A. Altomore, G. Cascarano, C. Giacovazzo, A. Guagliardi, M. C. Burla, G. Polidori and M. Camalli, *J. Appl. Cryst.*, **1994**, *27*, 435.
[2] P. W. Betteridge, J. R. Carruthers, R. I. Cooper, K. Prout and D. J. Watkin, *J. Appl. Cryst.*, **2003**, *36*, 1487.
[3] L. Palatinus and G. Chapuis, *J. Appl. Cryst.*, **2007**, *40*, 786-790.
[4] L. Palatinus, S. J. Prathapa and S. van Smaalen, *J. Appl. Cryst.,* **2012**, *45*, 575-580.
[5] G. M. Sheldrick, *Acta Cryst.*, **2008**, *A64*, 112-122.
